# Supplementary material for: Disentangling Electronic and Lattice Contributions to Transient Absorption in Metal Halide Perovskites: A First-Principles Study of CH3NH3PbBr3
Source: J Am Chem Soc. 2026 Sep 22;148(40):43632–40. doi: 10.1021/jacs.6c14114 (PMC13674265; doi:10.1021/jacs.6c14114)
Supplement: Supplementary file 1 [file ja6c14114_si_001.pdf]

Supporting Information for Disentangling Electronic and Lattice Contributions to Transient Absorption in Metal Halide Perovskites: A First-Principles Study of $CH_3NH_3PbBr_3$

Lu Qiao*[1], Ronaldo Rodrigues Pela[2], and Claudia Draxl[1,3]

[1]Department of Physics and CSMB, Humboldt-Universität zu Berlin, Berlin, Germany
[2]Distributed Algorithms and Supercomputing Department, Berlin, Germany
[3]European Theoretical Spectroscopy Facility (ETSF)

*Email: qiaolu@physik.hu-berlin.de

S1 Theoretical formalism for simulating pump-probe spectra

- **Step 1: DFT ground-state**

  The ground-state Kohn-Sham (KS) eigenvalues and orbitals obtained from density-functional theory provide the starting point for both the RT-TDDFT propagation and the subsequent BSE calculations.

- **Step 2: Construction of photoexcited carrier distributions via two routes**

  Photoexcited carrier distribution on the femtosecond timescale. The femtosecond-resolved evolution of the photoexcited carrier distribution is obtained from RT-TDDFT simulations. The pump pulse is introduced through a time-dependent vector potential $\mathbf{A}(t)$. Within this formulation, the time-dependent KS Hamiltonian is written as:[1]

$$\hat{H}(\mathbf{r},t) = \frac{1}{2}\left(-\mathrm{i}\nabla + \frac{1}{\mathrm{c}}\mathbf{A}(t)\right)^2 + V_{\mathrm{KS}}(\mathbf{r},t), \tag{S1}$$

  Starting from the ground-state orbitals, $\psi_{n\mathbf{k}}(\mathbf{r},t=0) = \psi^0_{n\mathbf{k}}(\mathbf{r})$ and their occupations $f^0_{n\mathbf{k}}$, the KS orbitals are propagated according to the time-dependent KS equation,

$$i\,\frac{\partial}{\partial t}\,\psi_{n\mathbf{k}}(\mathbf{r},t) = \hat{H}(\mathbf{r},t)\,\psi_{n\mathbf{k}}(\mathbf{r},t). \tag{S2}$$

  The time-dependent carrier distributions are then obtained by projecting the propagated orbitals onto the ground-state KS eigenstates $\{\psi^0_{c\mathbf{k}}, \psi^0_{v\mathbf{k}}\}$. The occupation of a conduction-band state $c\mathbf{k}$ is evaluated as

$$f_{c\mathbf{k}}(t) = \sum_n f^0_{n\mathbf{k}} \left|\langle \psi^0_{c\mathbf{k}} \,|\, \psi_{n\mathbf{k}}(t)\rangle\right|^2, \tag{S3}$$

  the hole distribution associated with a valence-band state $v\mathbf{k}$ is obtained from

$$f_{v\mathbf{k}}(t) = f^0_{v\mathbf{k}} - \sum_n f^0_{n\mathbf{k}} \left|\langle \psi^0_{v\mathbf{k}} \,|\, \psi_{n\mathbf{k}}(t)\rangle\right|^2. \tag{S4}$$

  Photoexcited carrier distribution on the picosecond timescale. At picosecond delays following photoexcitation, the carrier distribution is described within constrained density functional theory (cDFT). The hot carriers thermalize on subpicosecond timescales due to carrier-carrier and carrier-phonon scattering. Therefore, photoexcited electrons and holes are assumed to relax to states near the

conduction-band minimum (CBM) and valence-band maximum (VBM). The resulting carrier distribution is then represented by a Fermi-Dirac distribution at an electronic temperature T:

$$f_{c\mathbf{k}} = f^{\mathrm{e}}_{c\mathbf{k}} = \frac{1}{1+\exp\left[\dfrac{\varepsilon_{c\mathbf{k}}-\mu}{k_{\mathrm{B}}T}\right]}, \qquad f_{v\mathbf{k}} = f^{0}_{v\mathbf{k}} - f^{\mathrm{h}}_{v\mathbf{k}} = f^{0}_{v\mathbf{k}} - \frac{1}{1+\exp\left[\dfrac{\mu-\varepsilon_{v\mathbf{k}}}{k_{\mathrm{B}}T}\right]}. \tag{S5}$$

Here, k_{B} is the Boltzmann constant, μ is the Fermi level, and $f^{\mathrm{e}}_{c\mathbf{k}}$ and $f^{\mathrm{h}}_{v\mathbf{k}}$ denote the electron and hole occupations in the conduction and valence states, respectively. They are constrained to preserve charge neutrality and to reproduce the prescribed number of photoexcited carriers per unit cell, N_{exc}:

$$\sum_{\mathbf{k}} w_{\mathbf{k}}\, f^{\mathrm{e}}_{c\mathbf{k}} = \sum_{\mathbf{k}} w_{\mathbf{k}}\, f^{\mathrm{h}}_{v\mathbf{k}} = N_{\mathrm{exc}}, \tag{S6}$$

where $w_{\mathbf{k}}$ denotes the Brillouin-zone weight of each $\mathbf{k}$ point.

- **Step 3: Non-equilibrium extension of the BSE**

  The BSE is formulated as an effective two-particle eigenvalue problem,

$$\sum_{v'c'\mathbf{k}'} H^{\mathrm{BSE}}_{vc\mathbf{k},v'c'\mathbf{k}'}\, A^{\lambda}_{v'c'\mathbf{k}'} = E_{\lambda}\, A^{\lambda}_{vc\mathbf{k}}. \tag{S7}$$

  The BSE Hamiltonian matrix consists of three terms,

$$H^{\mathrm{BSE}} = H^{\mathrm{diag}} + 2H^{\mathrm{x}} + H^{\mathrm{dir}}, \tag{S8}$$

  where H^{diag}, H^{x}, and H^{dir} describe independent-particle transitions, bare electron-hole exchange, and screened electron-hole attraction, respectively. The screened Coulomb interaction $W_{\mathbf{GG}'}(\mathbf{q})$ entering H^{dir} is given by

$$W_{\mathbf{GG}'}(\mathbf{q}) = v_{\mathbf{G}}(\mathbf{q})\, \epsilon^{-1}_{\mathbf{GG}'}(\mathbf{q},\omega=0). \tag{S9}$$

  The microscopic dielectric matrix $\epsilon^{-1}_{\mathbf{GG}'}$ is evaluated within the random-phase approximation (RPA) from the independent-particle polarizability χ^0. To incorporate the effect of photoexcitation, χ^0 is computed by replacing the equilibrium occupations with the photoexcited carrier distributions obtained in Step 2:

$$\chi^{0}_{\mathbf{GG}'}(\mathbf{q},\omega) = \sum_{vc\mathbf{k}} \frac{f_{v\mathbf{k}} - f_{c,\mathbf{k}+\mathbf{q}}}{\omega - \left(\varepsilon^{0}_{c,\mathbf{k}+\mathbf{q}} - \varepsilon^{0}_{v\mathbf{k}}\right) + \mathrm{i}\eta}\, M^{\mathbf{G}}_{vc}(\mathbf{k},\mathbf{q}) \left[M^{\mathbf{G}'}_{vc}(\mathbf{k},\mathbf{q})\right]^{*}, \tag{S10}$$

  where $M^{\mathbf{G}}_{vc}(\mathbf{k},\mathbf{q}) = \langle \psi^{0}_{v\mathbf{k}} | e^{\mathrm{i}(\mathbf{q}+\mathbf{G})\cdot\mathbf{r}} | \psi^{0}_{c,\mathbf{k}+\mathbf{q}} \rangle$ denotes the planewave matrix element.

- **Step 4: Calculation of absorption spectra**

  The macroscopic dielectric function $\varepsilon_M(\omega)$ is obtained as:[2]

$$\varepsilon_M(\omega) = 1 + \frac{8\pi}{\Omega} \sum_{\lambda} \frac{|t_{\lambda}(\mathbf{q}\to 0)|^2}{E_{\lambda} - \omega - i\eta}, \tag{S11}$$

  where Ω is the unit-cell volume, and $t_{\lambda}(\mathbf{q}\to 0)$ is the transition coefficient:

$$t_{\lambda}(\mathbf{q}) = \sum_{vc\mathbf{k}} A^{\lambda}_{vc\mathbf{k}} \sqrt{f_{v\mathbf{k}} - f_{c,\mathbf{k}+\mathbf{q}}}\, \frac{\left\langle v\mathbf{k} \middle| \hat{p} \middle| c,\mathbf{k}+\mathbf{q} \right\rangle}{\varepsilon^{0}_{c,\mathbf{k}+\mathbf{q}} - \varepsilon^{0}_{v\mathbf{k}}}. \tag{S12}$$

  The absorption coefficient $\alpha(\omega)$ is derived from the complex macroscopic dielectric function $\varepsilon_M(\omega)$.[3]

- **Step 5: Disentanglement of electronic and lattice contributions to the transient spectra**

  Electronic contribution. The electronic contribution to the non-equilibrium absorption spectrum is isolated by solving the BSE using the photoexcited carrier distribution. The electronic transient

absorption spectrum at a pump-probe delay t is defined as the difference between the non-equilibrium spectrum, $\alpha^{\mathrm{neq}}(\omega, t)$, and the equilibrium spectrum, $\alpha^{\mathrm{eq}}(\omega)$:

$$\Delta\alpha(\omega, t) = \alpha^{\mathrm{neq}}(\omega, t) - \alpha^{\mathrm{eq}}(\omega). \tag{S13}$$

It contains contributions from two many-body effects: (i) *Pauli blocking*, arising from occupation-induced suppression of optical transitions, and (ii) *photoinduced Coulomb screening*, representing the modification of the electron-hole interaction due to the screening by photoexcited carriers. We disentangle the two contributions by selectively introducing the photoexcited carrier distribution either in the transition coefficients through the factor $\sqrt{f_{v\mathbf{k}} - f_{c,\mathbf{k}+\mathbf{q}}}$ (Step 4, Eq. S12) to isolate *Pauli blocking*, or in the construction of the statically screened Coulomb interaction (Step 3, Eq. S9–S10) to isolate *photoinduced Coulomb screening*.

Lattice contribution. The lattice contribution becomes important on the picosecond timescale, after a substantial fraction of the excess electronic energy is transferred to the lattice through carrier-phonon coupling and subsequent lattice relaxation. The pump-induced lattice response is described by two contributions: (i) *lattice expansion* and (ii) *lattice vibrations*, which introduce dynamic lattice disorder in the structure.

To account for the lattice expansion, we adopt a crystal structure reflecting expansion at a given temperature T and compute the corresponding absorption spectrum, denoted as $\alpha^{\mathrm{LE}}(\omega, T)$. The transient absorption associated with lattice expansion is then defined as:

$$\Delta\alpha^{\mathrm{LE}}(\omega, T) = \alpha^{\mathrm{LE}}(\omega, T) - \alpha^{\mathrm{eq}}(\omega), \tag{S14}$$

where $\alpha^{\mathrm{eq}}(\omega)$ is the equilibrium spectrum obtained for the ground-state structure.

The contribution from lattice vibrations is treated by *ab initio* molecular dynamics (AIMD). Within the Born-Oppenheimer approximation, dynamic lattice disorder is represented by an ensemble of a number (N) of instantaneous ionic configurations $\mathbf{R}_j(T)$ sampled from a finite-temperature AIMD trajectory. KS eigenvalues, orbitals, and screened electron-hole interaction are functions of the ionic coordinates. For each configuration j, the BSE is solved, yielding $\alpha_j(\omega, T)$. The spectrum reflecting lattice vibrations, $\alpha^{\mathrm{LV}}(\omega, T)$, is then obtained by averaging over them:

$$\alpha^{\mathrm{LV}}(\omega, T) \approx \frac{1}{N} \sum_{j=1}^{N} \alpha_j(\omega, T). \tag{S15}$$

The transient absorption associated with lattice vibrations is then defined as:

$$\Delta\alpha^{\mathrm{LV}}(\omega, T) = \alpha^{\mathrm{LV}}(\omega, T) - \alpha^{\mathrm{eq}}(\omega). \tag{S16}$$

S2 Computational details

S2.1 Geometry optimization

The atomic positions of the cubic $CH_3NH_3PbBr_3$ are relaxed using the experimental lattice parameter of 5.98 Å[4] with the FHI-aims package.[5] The PBE functional is employed for exchange-correlation effects.[6] Brillouin-zone (BZ) integrations are performed with an $8 \times 8 \times 8$ Monkhorst-Pack **k**-point.

S2.2 Ground-state calculations

All calculations are carried out within the all-electron full-potential code `exciting`, based on the linearized augmented planewave plus local-orbital (LAPW+LO) method.[7,8] The Perdew-Burke-Ernzerhof functional is used as the exchange-correlation functional.[6] Ground-state DFT calculations are performed using an $8\times8\times8$ Monkhorst–Pack **k**-point grid for Brillouin-zone sampling.[9] The LAPW basis-set cutoff is set to 4.14 a_0^{-1}, which corresponds to an $R_{\mathrm{MT}}|\mathbf{G}+\mathbf{k}|_{\max}$ value of 2.9 for the smallest muffin-tin radius of $0.7\,a_0$ used for hydrogen. The other muffin-tin radii are chosen as $R_{\mathrm{C}} = 1.2\,a_0$, $R_{\mathrm{N}} = 1.2\,a_0$, $R_{\mathrm{Pb}} = 2.5\,a_0$, and $R_{\mathrm{Br}} = 2.5\,a_0$. Spin-orbit coupling is included using the second-variational scheme.[10]

S2.3 Excited-state calculations

Table S1 shows the computational parameters employed in the cDFT, RT-TDDFT, and BSE calculations. Pump photon energy (E_{hv}), polarization, and pulse duration specify the frequency, orientation, and temporal profile of the external field. Local-field effects are included up to a cutoff of $|\mathbf{G}+\mathbf{q}|_{\max}$. A life-time broadening with a Lorentzian linewidth is applied to the spectra. N_{empty} is the number of unoccupied orbitals included in the BSE calculations. Scissor corrections are used to align the calculated equilibrium spectra with the experimental counterparts. The same values are then applied to all spectra.

Table S1: Computational parameters used in cDFT, RT-TDDFT, and BSE calculations.

Method	Parameter	X-ray region	Optical region
All	$\mathbf{k}$-mesh	$4 \times 4 \times 4$	$8 \times 8 \times 8$
	$R_{\text{MT}}|\mathbf{G}+\mathbf{k}|_{\max}$	2.9	2.9
RT-TDDFT	Photon energy (eV)	3.1	3.1
	Polarization	x	x
	Pulse duration (fs)	1	1
BSE	$|\mathbf{G}+\mathbf{q}|_{\max}$ (a.u.$^{-1}$)	2.0	2.0
	Broadening (eV)	1.9	0.095
	N_{empty}	346	10 VB, 10 CB
	Scissor shift (eV)	161.5	1.23

S2.4 Expanded lattice

To account for lattice expansion, the lattice parameter of $CH_3NH_3PbBr_3$ is evaluated at the experimentally reported pump-induced temperature of 373 K.[11] Based on the experimental lattice parameter at room temperature, $a = 5.98$ Å,[4] and a thermal expansion coefficient of 2.5×10^{-4} Å K^{-1},[12] we obtain a value of $a \approx 6.00$ Å at 373 K, which is used to construct the thermally expanded structure for subsequent BSE calculations. Figure S1 shows the effects of photoexcited carriers and lattice expansion on the density of states.

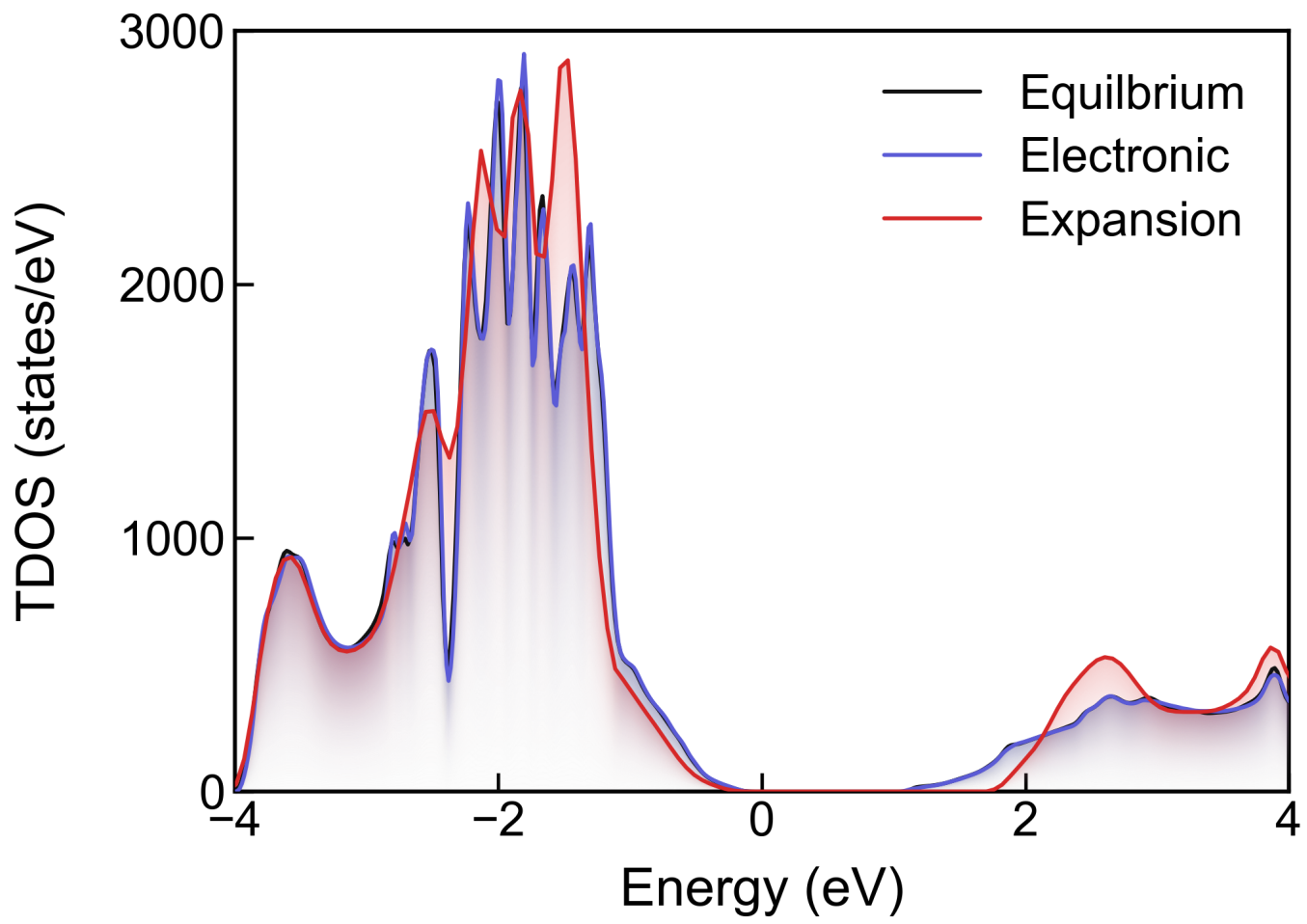

Figure S1: Total density of states for the equilibrium (black area), photoexcited (blue area), and expanded-lattice (red area) systems.

S2.5 AIMD sampling

Lattice vibrations are sampled by AIMD simulations performed with the Vienna *Ab initio* Simulation Package (VASP)[13,14] on a $2\times2\times2$ cubic supercell of $CH_3NH_3PbBr_3$.[15–17] The AIMD simulations are first carried out in the NVE ensemble for 8 ps, during which the temperature equilibrates to approximately 373 K, followed by a 12 ps NVT simulation at 373 K using a Nosé-Hoover thermostat. A total of 30 snapshots are extracted from the equilibrated NVT trajectory at intervals of 400 fs and used as input geometries for subsequent BSE calculations. The absorption spectrum incorporating the effects of lattice vibrations is obtained by averaging the spectra calculated for all snapshots. Figure S2 depicts the density of states for the snapshots to show the impact of the vibrations.

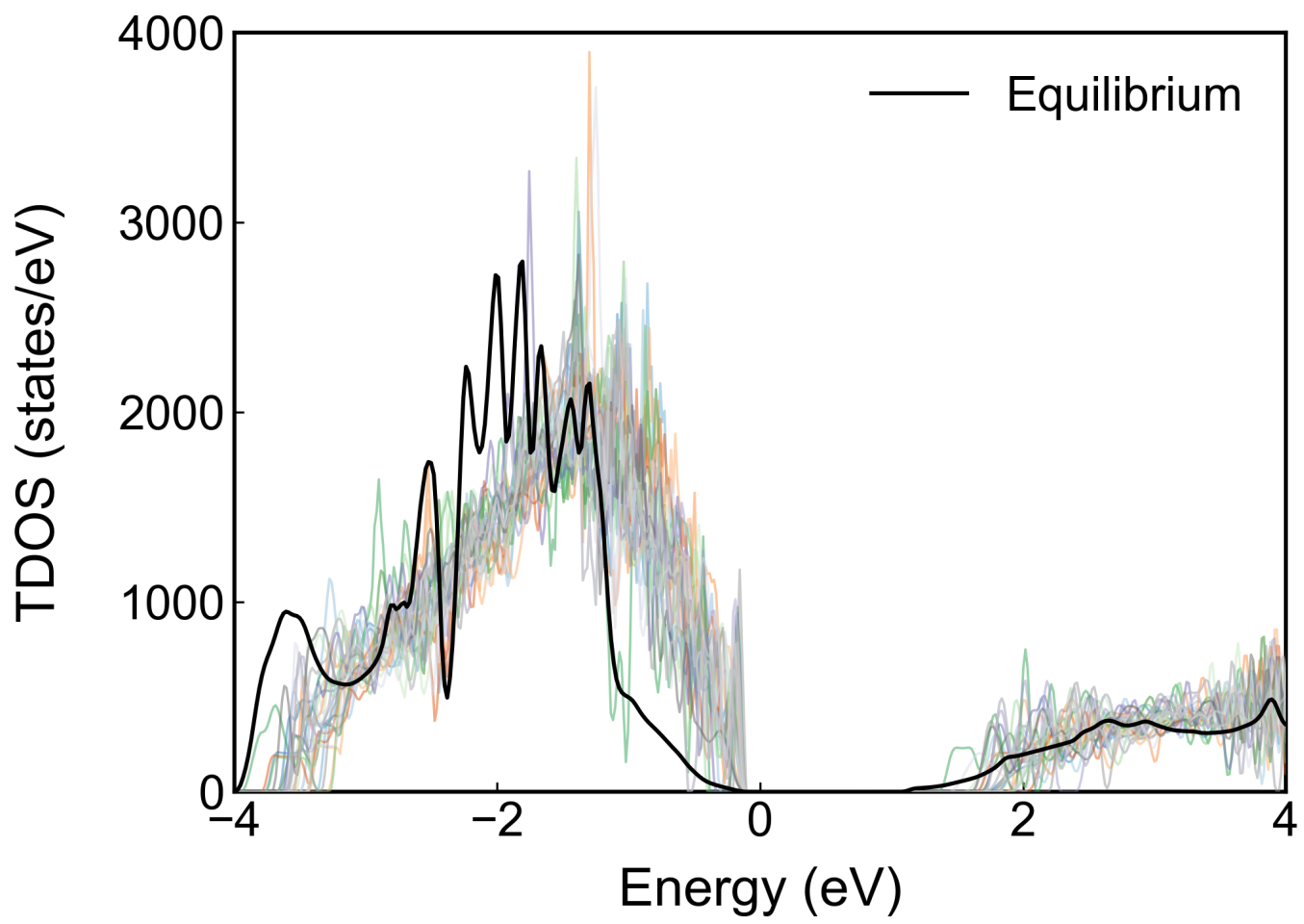

Figure S2: Total density of states for the equilibrium system (black curve) and representative AIMD snapshots (colored curves).

To evaluate the convergence of the vibrationally averaged spectra with respect to the number of AIMD snapshots, we performed a random-subsampling analysis for both the X-ray and optical absorption spectra. From the complete set of 30 snapshots, subsets containing $N = 5, 10, 15, 20$, and 25 snapshots were randomly selected without replacement, with 500 independent subsets generated for each $N < 30$. The spectrum averaged over all 30 snapshots was taken as the reference, and the deviation of each subset-averaged spectrum was quantified by the normalized root-mean-square deviation (NRMSD),

$$\mathrm{NRMSD}_N = \sqrt{\frac{\sum_E \left[\bar{\alpha}_N(E) - \bar{\alpha}_{30}(E)\right]^2}{\sum_E \bar{\alpha}_{30}(E)^2}}. \tag{S17}$$

For each N, the spectrum whose NRMSD was closest to the median value of the 500 realizations was selected as the representative spectrum. The resulting convergence tests are shown in the upper panels of Figs. S3 and S4 for the X-ray and optical absorption spectra, respectively. In both spectral regions, the spectra obtained with $N = 20$, 25, and 30 snapshots are nearly indistinguishable over the entire energy range, indicating that the vibrationally averaged spectrum is already statistically converged with approximately 20 snapshots.

We additionally quantified the spectral variation among different AIMD snapshots by calculating the standard deviation of the absorption intensity at each energy point over the 30 individual spectra

$$\sigma_\alpha(E) = \sqrt{\frac{1}{N-1}\sum_{j=1}^{N}\left[\alpha_j(E) - \bar{\alpha}(E)\right]^2}, \qquad N = 30. \tag{S18}$$

Here, $\alpha_j(E)$ denotes the absorption spectrum obtained from the jth AIMD snapshot, and $\bar{\alpha}(E)$ is the spectrum averaged over all 30 snapshots. The lower panels of Figs. S3 and S4 show the 30-snapshot averaged

spectra together with the corresponding $\pm\sigma_\alpha(E)$ regions. The shaded regions characterize the spectral variation among different AIMD snapshots.

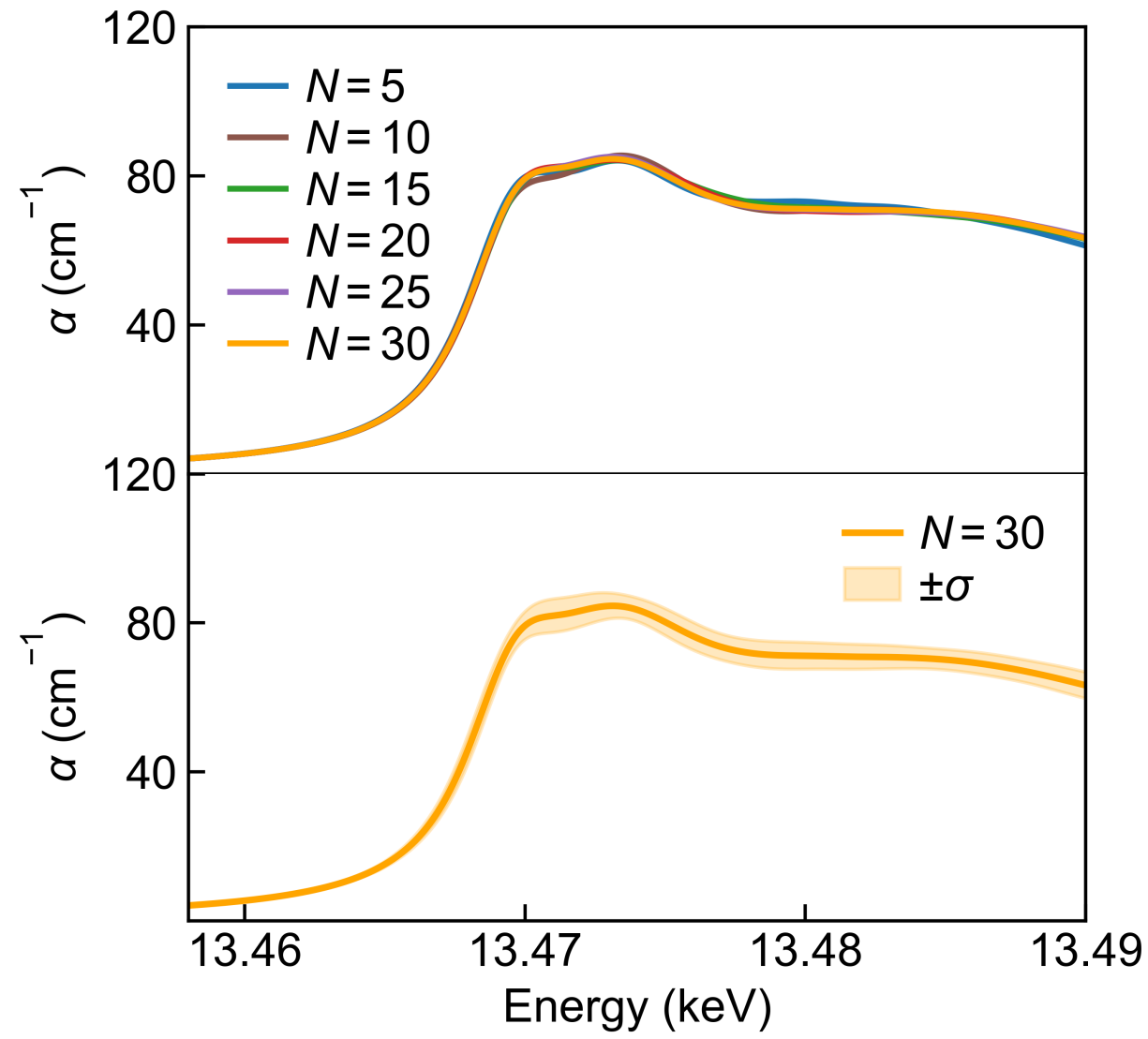

Figure S3: Convergence and spectral variation of the vibrationally averaged X-ray absorption spectrum at the Br K-edge of $CH_3NH_3PbBr_3$. Top: Representative spectra obtained using $N = 5, 10, 15, 20, 25$, and 30 AIMD snapshots. Bottom: Spectrum averaged over all 30 AIMD snapshots (orange curve). The shaded region represents the 30-snapshot average $\pm\sigma_\alpha(E)$, where $\sigma_\alpha(E)$ is the standard deviation of the absorption intensity across the 30 AIMD snapshots at each energy point.

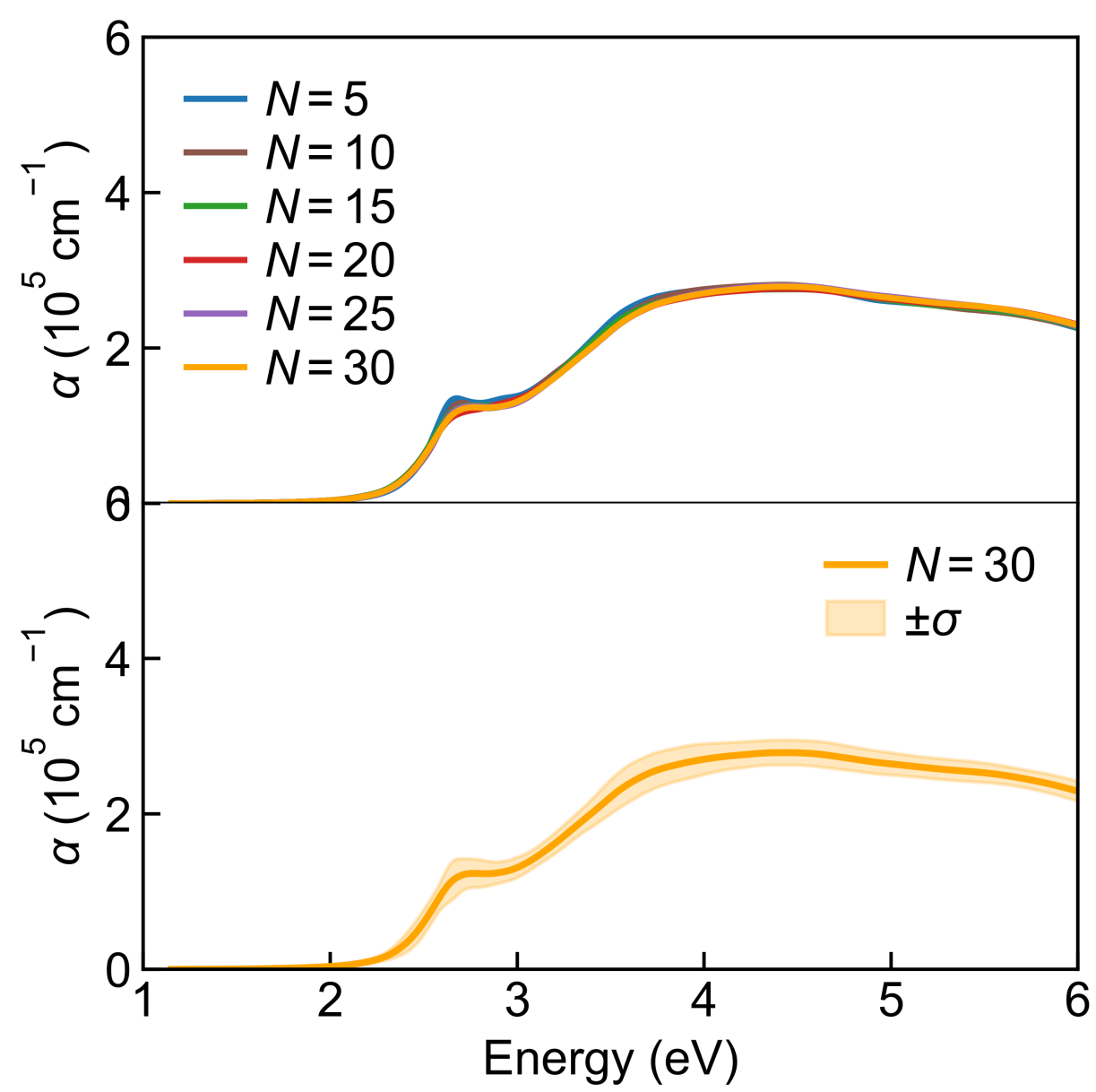

Figure S4: Convergence and spectral variation of the vibrationally averaged optical absorption spectrum in $CH_3NH_3PbBr_3$. The analysis is the same as that in Fig. S3, but for the optical region.

S3 Carrier-induced band-gap renormalization

To evaluate the contribution of carrier-induced band-gap renormalization (BGR), we calculated the X-ray and optical absorption spectra within the independent-particle approximation (IPA) at an excitation density of $n_e = 3.0 \times 10^{20}$ cm^{-3}, using the photoexcited-state electronic structure while retaining the equilibrium occupations. In the IPA, the electron-hole interaction kernel is absent, thereby excluding the contribution of photoinduced Coulomb screening, while the use of equilibrium occupations eliminates Pauli blocking. The difference between the photoexcited-state and equilibrium IPA spectra therefore predominantly reflects the carrier-induced BGR.

The BGR contribution to the transient spectra is smaller than those from Pauli blocking and photoinduced Coulomb screening in both the X-ray (Fig. S5) and optical regions (Fig. S6). In the X-ray region, the BGR contribution to transient spectrum is nearly two orders of magnitude smaller than the corresponding screening contribution. In the optical region, it remains approximately one order of magnitude smaller. It demonstrates that BGR can be safely neglected in the decomposition of the transient spectra.

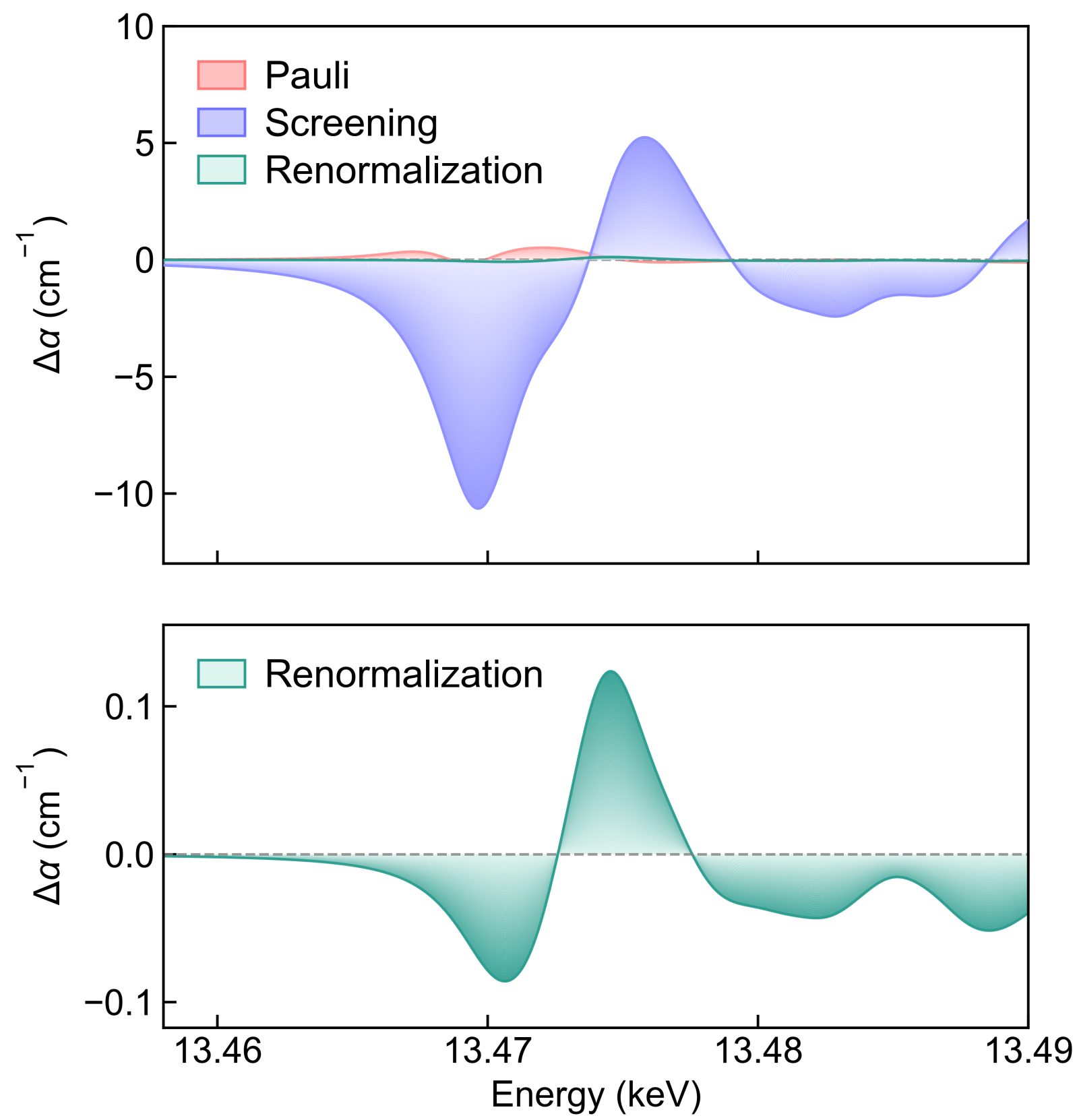

Figure S5: Top: Contributions of Pauli blocking (red area), photoinduced Coulomb screening (blue area), and carrier-induced band-gap renormalization (BGR, green area) to the XTA spectrum at the Br K-edge of $CH_3NH_3PbBr_3$ at an excitation density of $n_e = 3.0 \times 10^{20}$ cm^{-3}. Bottom: Enlarged view of the BGR contribution.

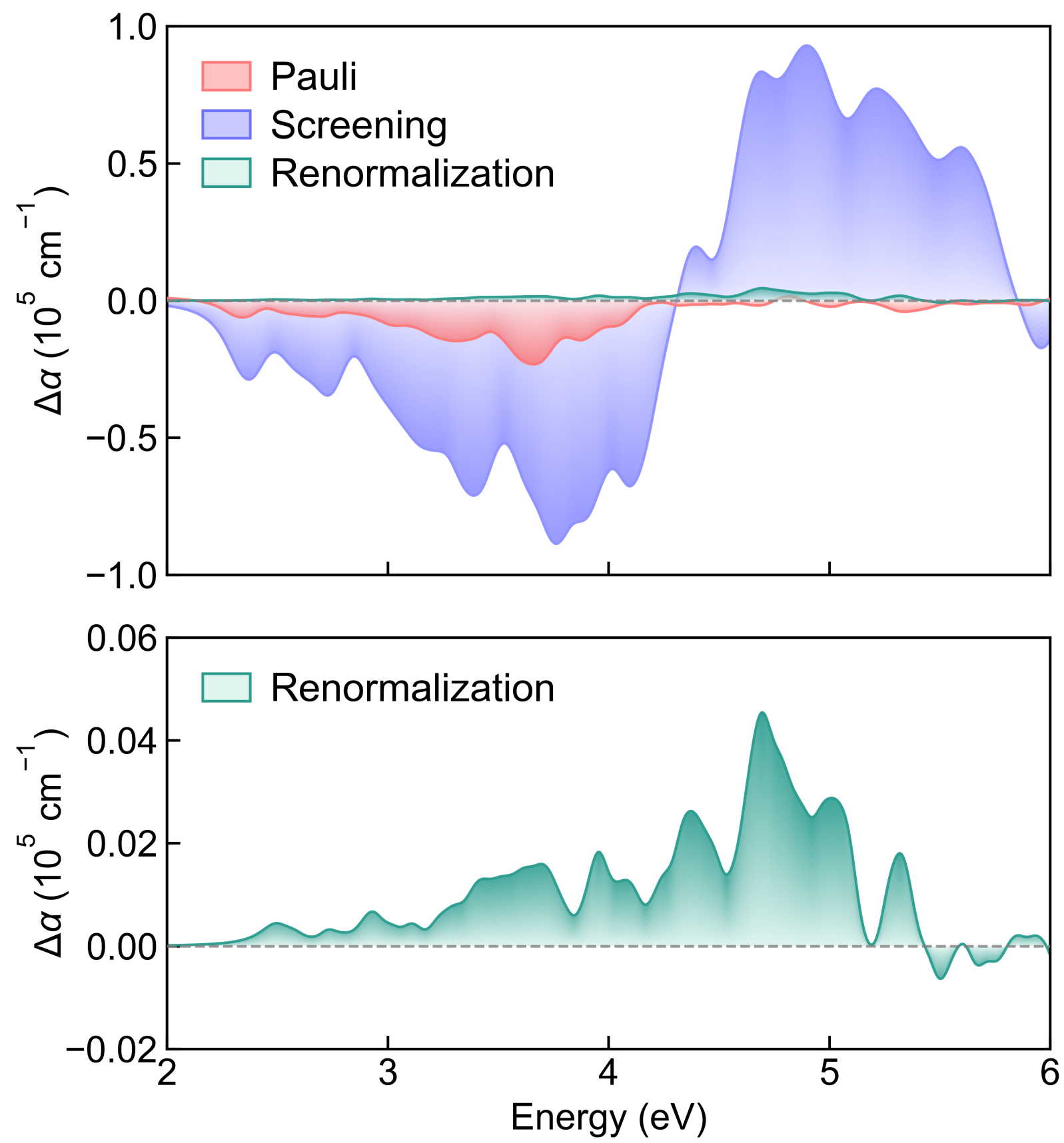

Figure S6: Top: Contributions of Pauli blocking (red area), photoinduced Coulomb screening (blue area), and carrier-induced band-gap renormalization (BGR, green area) to the optical TA spectrum of $CH_3NH_3PbBr_3$ at an excitation density of $n_e = 3.0 \times 10^{20}$ cm^{-3}. Bottom: Enlarged view of the BGR contribution.

S4 Decomposition of the photoinduced Coulomb screening contribution

To identify the spectral changes induced by photoinduced Coulomb screening, we decompose it by making use of the equilibrium absorption spectrum $\alpha(E)$ and its first two energy derivatives:[18]

$$\Delta\alpha(E) \approx c_0\alpha(E) + c_1\frac{\partial \alpha}{\partial E} + c_2\frac{\partial^2 \alpha}{\partial E^2}. \tag{S19}$$

Here, c_0 accounts for changes in spectral intensity, c_1 captures the spectral shift, and c_2 describes redistributions of spectral weight.

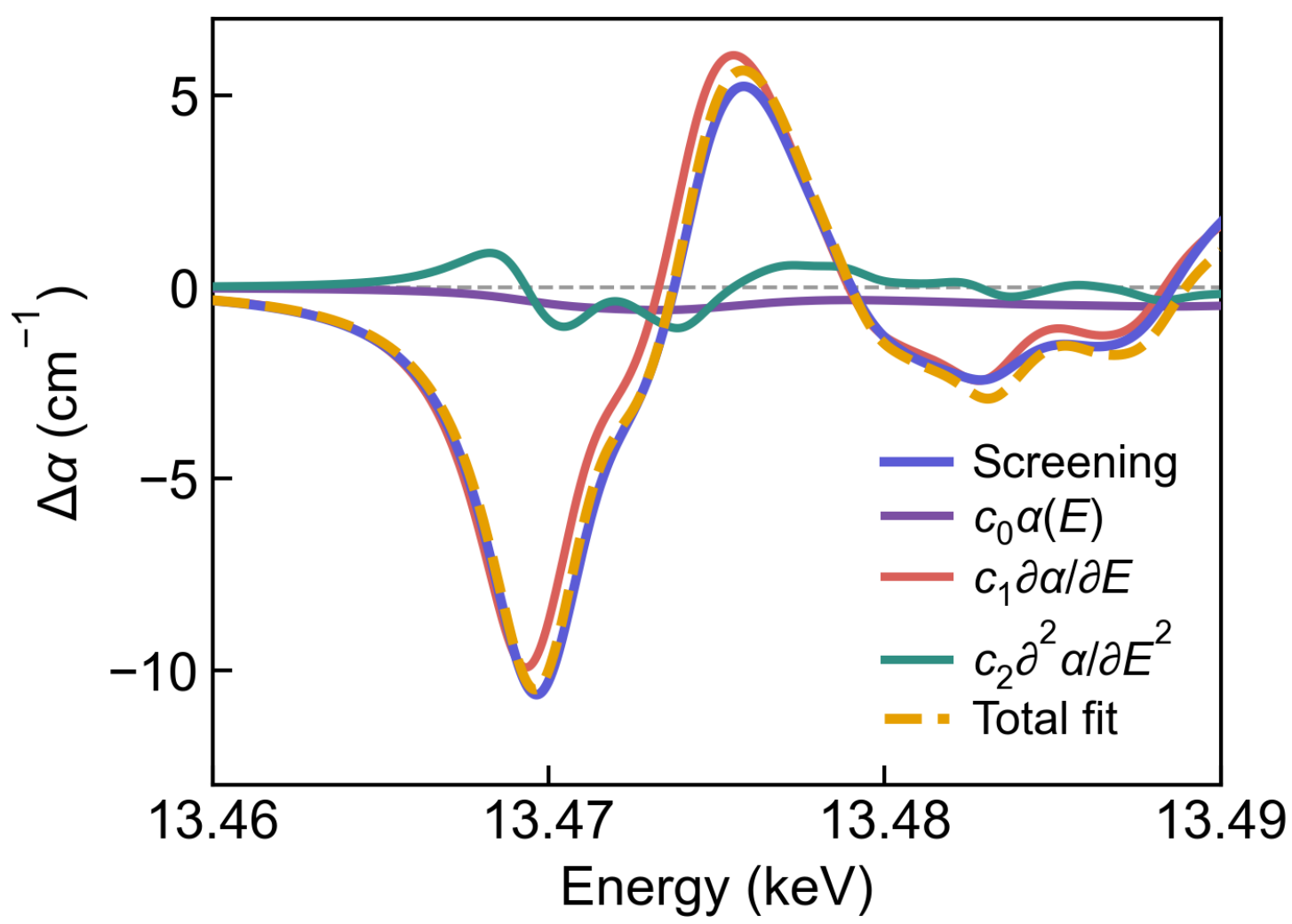

Figure S7: Contributions to the XTA spectrum on the femtosecond timescale obtained by fitting it with the equilibrium absorption spectrum and its energy derivatives.

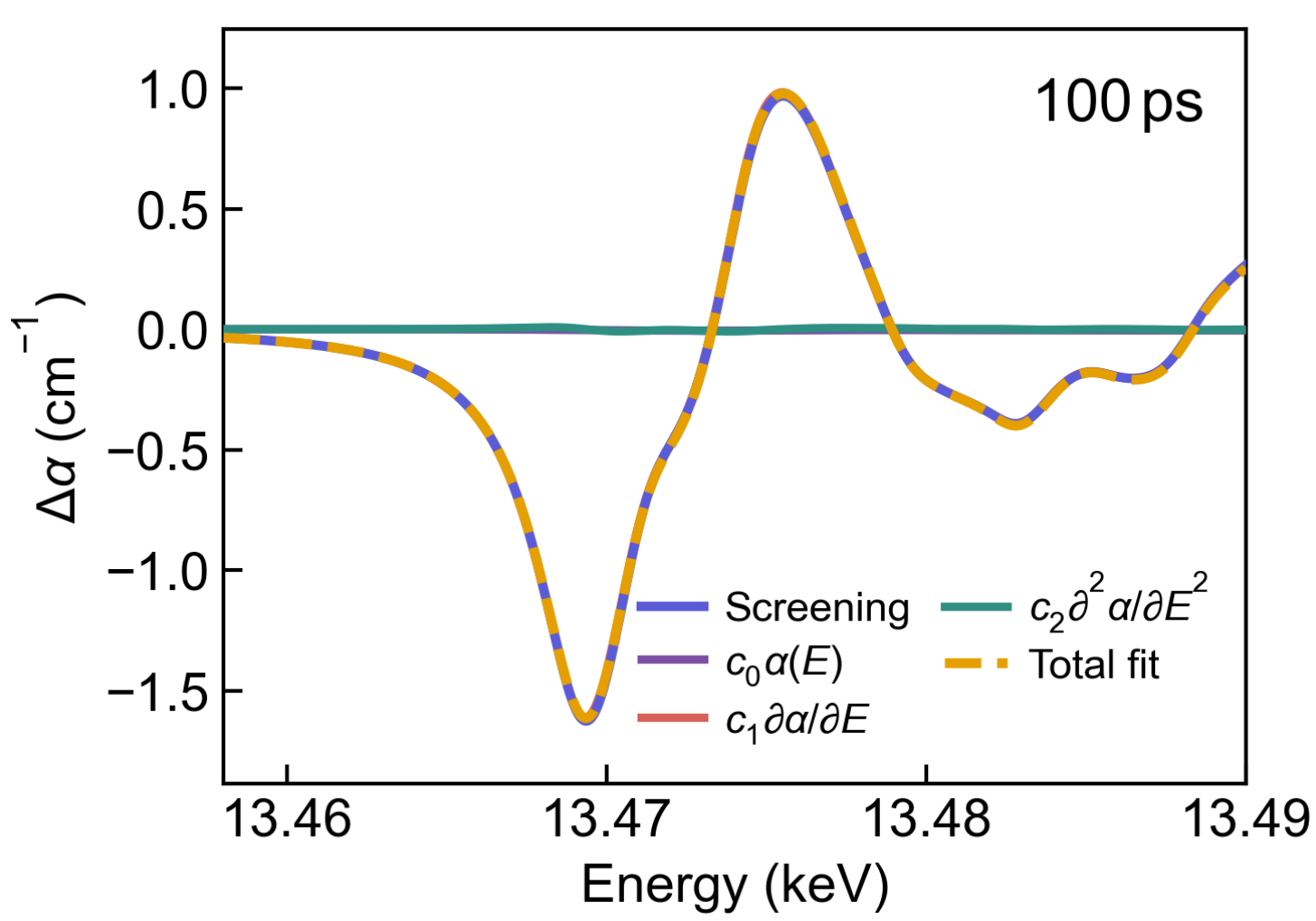

Figure S8: Contributions to the XTA spectrum on the picosecond timescale obtained by fitting it with the equilibrium absorption spectrum and its energy derivatives.

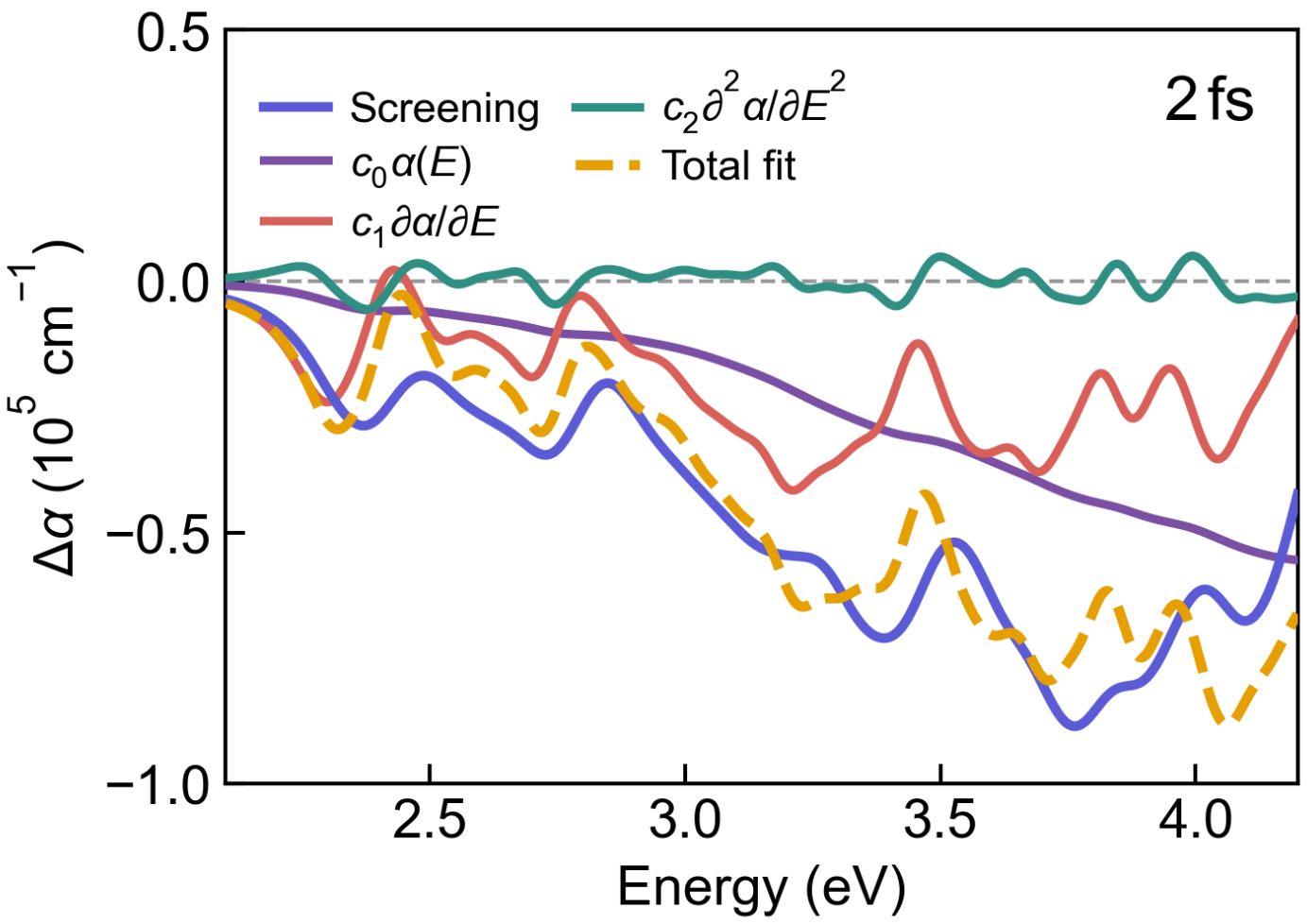

Figure S9: Contribution to the optical TA spectrum on the femtosecond timescale obtained by fitting it with the equilibrium absorption spectrum and its energy derivatives.

Figures S7 and S8 show that the total fitted spectra closely reproduce the screening contribution to the XTA spectra on the femtosecond and picosecond timescales, respectively. The first-derivative term dominates in both cases, while the zeroth- and second-derivative contributions are much smaller, particularly on the picosecond timescale. This indicates that the photoinduced Coulomb screening mainly induces a spectral shift in the XTA spectra. Figure S9 shows that both zeroth- and first-derivative terms contribute to the optical TA spectrum on the femtosecond timescale, while the second-derivative contribution remains small, indicating that photoinduced Coulomb screening leads to an intensity reduction and a spectral shift.

S5 Spectral moments

Spectral moments have long been used to connect spectral lineshapes to lattice motion and provide a compact framework for comparing spectra obtained from different structural configurations and thermal conditions.[19] Specifically, they provide quantitative descriptors for the spectral lineshape, including the absorption intensity, average excitation energy, spectral width, and asymmetry.[19]

The zeroth spectral moment, M_0, is obtained by integrating the spectral intensity over the selected energy range, which reflects the overall absorption intensity across the spectral region of interest.

$$M_0 = \int I(E)\,\mathrm{d}E. \tag{S20}$$

For a discrete absorption spectrum composed of transitions with excitation energies E_i and spectral intensities $I(E_i)$, the first spectral moment, $\bar{E}$, is obtained by weighting each transition according to its contribution to the total absorption intensity. This quantity therefore represents the intensity-weighted average excitation energy, analogous to the center of mass of the spectral distribution.

$$\bar{E} = \frac{\sum_i E_i I(E_i)\Delta E_i}{\sum_i I(E_i)\Delta E_i}. \tag{S21}$$

Taking the limit of an infinitely fine energy discretization gives:

$$\bar{E} = \lim_{\Delta E_i \to 0} \frac{\sum_i E_i I(E_i)\Delta E_i}{\sum_i I(E_i)\Delta E_i} = \frac{\int E I(E)\,dE}{\int I(E)\,dE}. \tag{S22}$$

The second spectral moment, σ^2, is obtained by weighting the squared deviation of each transition energy from the average excitation energy, $\bar{E}$, according to its contribution to the total absorption intensity. This

quantity represents the intensity-weighted variance of the spectral distribution and characterizes the spread of spectral weight around the average excitation energy:

$$\sigma^2 = \frac{\int (E - \bar{E})^2 I(E)\,\mathrm{d}E}{\int I(E)\,\mathrm{d}E}, \tag{S23}$$

The square root of this quantity, σ, provides a direct measure of the spectral width. A larger σ indicates a broader spectral weight distribution.

The third spectral moment, γ_1, is obtained by weighting the cubed deviation of each transition energy from the average excitation energy, $\bar{E}$. This quantity characterizes the asymmetry of the spectral distribution around the average excitation energy.

$$\gamma_1 = \frac{\int (E - \bar{E})^3 I(E)\,\mathrm{d}E}{\left(\int I(E)\,\mathrm{d}E\right)\sigma^3}. \tag{S24}$$

The normalization by σ^3 makes γ_1 dimensionless and enables spectra with different widths to be compared on the same scale. The factor $(E - \bar{E})^3$ preserves the sign of the deviation from the centroid, spectral weight on the high- and low-energy sides contributes with opposite signs. A positive γ_1 indicates a more pronounced high-energy tail, whereas a negative γ_1 indicates a stronger low-energy tail. A value close to zero corresponds to an approximately symmetric spectral profile around $\bar{E}$. Thus, γ_1 provides a measure of asymmetric broadening and spectral-weight redistribution.

References

[1] Yabana, K.; Sugiyama, T.; Shinohara, Y.; Otobe, T.; Bertsch, G. F. Time-Dependent Density Functional Theory for Strong Electromagnetic Fields in Crystalline Solids. *Phys. Rev. B* **2012**, *85*, 045134.

[2] Albrecht, S.; Reining, L.; Del Sole, R.; Onida, G. Ab Initio Calculation of Excitonic Effects in the Optical Spectra of Semiconductors. *Phys. Rev. Lett.* **1998**, *80*, 4510.

[3] Ambrosch-Draxl, C.; Sofo, J. O. Linear Optical Properties of Solids within the Full-Potential Linearized Augmented Planewave Method. *Comput. Phys. Commun.* **2006**, *175*, 1–14.

[4] Lei, Y.; Chen, Y.; Gu, Y.; Wang, C.; Huang, Z.; Qian, H.; Nie, J.; Hollett, G.; Choi, W.; Yu, Y.; others Controlled Homoepitaxial Growth of Hybrid Perovskites. *Adv. Mater.* **2018**, *30*, 1705992.

[5] Blum, V.; Gehrke, R.; Hanke, F.; Havu, P.; Havu, V.; Ren, X.; Reuter, K.; Scheffler, M. Ab Initio Molecular Simulations with Numeric Atom-Centered Orbitals. *Comput. Phys. Commun.* **2009**, *180*, 2175–2196.

[6] Perdew, J. P.; Burke, K.; Ernzerhof, M. Generalized Gradient Approximation Made Simple. *Phys. Rev. Lett.* **1996**, *77*, 3865.

[7] Gulans, A.; Kontur, S.; Meisenbichler, C.; Nabok, D.; Pavone, P.; Rigamonti, S.; Sagmeister, S.; Werner, U.; Draxl, C. exciting: A Full-Potential All-Electron Package Implementing Density-Functional Theory and Many-Body Perturbation Theory. *J. Phys.: Condens. Matter* **2014**, *26*, 363202.

[8] Raya-Moreno, M.; Buccheri, A.; Dasch, N. A.; Farahani, N.; Oliva, I. G.; Gulans, A.; Hossain, M.; Kleine, H.; Kuban, M.; Lubeck, S.; others An Exciting Approach to Theoretical Spectroscopy. *arXiv preprint arXiv:2601.11388* **2026**,

[9] Monkhorst, H. J.; Pack, J. D. Special Points for Brillouin-Zone Integrations. *Phys. Rev. B* **1976**, *13*, 5188.

[10] Vona, C.; Lubeck, S.; Kleine, H.; Gulans, A.; Draxl, C. Accurate and efficient treatment of spin-orbit coupling via second variation employing local orbitals. *Phys. Rev. B* **2023**, *108*, 235161.

[11] Wang, L.; Nughays, R.; Rossi, T. C.; Oppermann, M.; Ogieglo, W.; Bian, T.; Shih, C.-H.; Guo, T.-F.; Pinnau, I.; Yin, J.; others Disentangling Thermal from Electronic Contributions in the Spectral Response of Photoexcited Perovskite Materials. *J. Am. Chem. Soc.* **2024**, *146*, 5393–5401.

[12] Lang, A.; Polishchuk, I.; Seknazi, E.; Feldmann, J.; Katsman, A.; Pokroy, B. Bioinspired Molecular Bridging in a Hybrid Perovskite Leads to Enhanced Stability and Tunable Properties. *Adv. Funct. Mater.* **2020**, *30*, 2005136.

[13] Kresse, G.; Furthmüller, J. Efficiency of Ab-Initio Total Energy Calculations for Metals and Semiconductors Using a Plane-Wave Basis Set. *Comput. Mater. Sci.* **1996**, *6*, 15–50.

[14] Kresse, G.; Furthmüller, J. Efficient Iterative Schemes for Ab Initio Total-Energy Calculations Using a Plane-Wave Basis Set. *Phys. Rev. B* **1996**, *54*, 11169–11186.

[15] Dubajic, M.; Neilson, J. R.; Klarbring, J.; Liang, X.; Bird, S. A.; Rule, K. C.; Auckett, J. E.; Selby, T. A.; Tumen-Ulzii, G.; Lu, Y.; others Dynamic Nanodomains Dictate Macroscopic Properties in Lead Halide Perovskites. *Nat. Nanotechnol.* **2025**, 1–9.

[16] Garrote-Márquez, A.; Lodeiro, L.; Hernández, N. C.; Liang, X.; Walsh, A.; Menéndez-Proupin, E. Picosecond Lifetimes of Hydrogen Bonds in the Halide Perovskite $CH_3NH_3PbBr_3$. *J. Phys. Chem. C* **2024**, *128*, 20947–20956.

[17] Saleh, G.; Biffi, G.; Di Stasio, F.; Martín-García, B.; Abdelhady, A. L.; Manna, L.; Krahne, R.; Artyukhin, S. Methylammonium Governs Structural and Optical Properties of Hybrid Lead Halide Perovskites through Dynamic Hydrogen Bonding. *Chem. Mater.* **2021**, *33*, 8524–8533.

[18] Kulakov, T.; Paraschuk, D. First and Second Derivative Analysis of Electroabsorption Spectra in Conjugated Molecules and Polymers: Stark Shift and Stark Broadening. *Chem. Phys. Lett.* **2000**, *325*, 517–522.

[19] Gordon, R. G. Molecular Motion and the Moment Analysis of Molecular Spectra in Condensed Phases. I. Dipole-Allowed Spectra. *J. Chem. Phys.* **1963**, *39*, 2788–2797.
